# Post-Natal Inhibition of NF-κB Activation Prevents Renal Damage Caused by Prenatal LPS Exposure

**DOI:** 10.1371/journal.pone.0153434

**Published:** 2016-04-13

**Authors:** Wei Guo, Xiao Guan, Xiaodong Pan, Xiongshan Sun, Fangjie Wang, Yan Ji, Pei Huang, Yafei Deng, Qi Zhang, Qi Han, Ping Yi, Michael Namaka, Ya Liu, Youcai Deng, Xiaohui Li

**Affiliations:** 1 Institute of Materia Medica, College of Pharmacy, Third Military Medical University, Chongqing 400038, China; 2 Department of Pharmacy, Southwest Hospital, Third Military Medical University, Chongqing 400038, China; 3 Center of Translational Medicine, College of Pharmacy, Third Military Medical University, Chongqing 400038, China; 4 Department of Obstetrics and Gynecology, Daping Hospital, Third Military Medical University, Chongqing 400038, China; 5 Colleges of Pharmacy and Medicine, University of Manitoba, Apotex Center 750, McDermot Avenue, Winnipeg, R3E 0T5, MB, Canada; 6 Joint Laboratory of Biological Psychiatry between Shantou University Medical College and the College of Medicine University of Manitoba, Shantou 515063, China; University Medical Center Utrecht, NETHERLANDS

## Abstract

Prenatal exposure to an inflammatory stimulus has been shown to cause renal damage in offspring. Our present study explored the role of intra-renal NF-κB activation in the development of progressive renal fibrosis in offspring that underwent prenatal exposure to an inflammatory stimulus. Time-dated pregnant rats were treated with saline (control group) or 0.79 mg/kg lipopolysaccharide (LPS) through intra-peritoneal injection on gestational day 8, 10 and 12. At the age of 7 weeks, offspring from control or LPS group were treated with either tap water (Con+Ve or LPS+Ve group) or pyrollidine dithiocarbamate (PDTC, 120mg/L), a NF-κB inhibitor, *via* drinking water starting (Con+PDTC or LPS+PDTC group), respectively, till the age of 20 or 68 weeks. The gross structure of kidney was assessed by hematoxylin-eosin, periodic acid–Schiff staining and Sirius red staining. The expression levels of TNF-α, IL-6, α-smooth muscle actin (α-SMA) and renin-angiotensin system (RAS) genes were determined by real time polymerase chain reaction and/or immunohistochemical staining. Our data showed that post-natal persistent PDTC administration efficiently repressed intra-renal NF-κB activation, TNF-α and IL-6 expression. Post-natal PDTC also prevented intra-renal glycogen deposition and collagenous fiber generation as evident by the reduced expression of collagen III and interstitial α-SMA in offspring of prenatal LPS exposure. Furthermore, post-natal PDTC administration reversed the intra-renal renin-angiotensin system (RAS) over-activity in offspring of prenatal LPS exposure. In conclusion, prenatal inflammatory exposure results in offspring’s intra-renal NF-κB activation along with inflammation which cross-talked with excessive RAS activation that caused exacerbation of renal fibrosis and dysfunction in the offspring. Thus, early life prevention of NF-κB activation may be a potential preventive strategy for chronic renal inflammation and progressive renal damage.

## Introduction

The incidence and morbidity rates of cardiovascular disease (CVD) such as hypertension continue to rise despite ongoing research efforts in regard to primary and secondary prevention [1]. Therefore, exploring novel mechanisms yielding new biological targets are of great significance to improve prevention and treatment of hypertension as well as to reduce the incidence of CVD.

The association of adverse intra-uterine environment with adult chronic diseases in offspring has attracted significant attention worldwide [2]. Based on an epidemiological study in England, they showed that women with bacterial vaginosis had a fivefold increased risk of pre-term delivery independent of age, race, marital status, education, income and history of preterm birth [3]. The children born with pre-term birth showed the characteristic risk factors for the development of cardiovascular disease, such as higher blood pressure, higher fasting levels of serum free fatty acids [4]. A recent epidemiological study had also demonstrated that the population involved with the prenatal exposure to the 1918 influenza pandemic showed ~ 20% excess cardiovascular disease [4]. These findings provide supportive evidence that prenatal inflammation is epidemiologically relevant to CVD. We previously found that prenatal exposure to inflammatory stimuli, such as lipopolysaccharides (LPS), the main component of gram-negative bacteria cellular wall [5], resulted in the development of hypertension in Sprague-Dawley (SD) rats [6]. As such, this may be indirectly caused by maternal derived pro-inflammatory cytokines exposure, rather than directly LPS exposure in utero [7]. As prenatal inflammatory exposure, such as infection [8], hepatitis [9] as well as arthritis [10], is still the most common public health problems during pregnancy, further research is required in this area to uncover the mechanisms of prenatal programmed hypertension and other CVD complications.

Interestingly, our research group has shown that prenatal exposure to LPS resulted in significantly lower glomerular numbers, creatinine clearance rates and higher urinary protein in adult offspring [11]. Our reported findings in this area was also confirmed by another independent research group [12]. Mechanistic studies found that abnormality of intra-renal renin-angiotensin system (RAS) [11] and oxidative stress [12] might be involved. However, the relative mechanisms of prenatal LPS exposure induced renal damage are still largely unknown.

We previously found an increased renal infiltrating monocytes/macrophages and lymphocytes at the age of 7 weeks [13], and also an intra-renal NF-κB activation in offspring of prenatal exposure to LPS at the age of 25 weeks [11]. A specific IκBα degradation inhibitor, pyrollidine dithiocarbamate (PDTC) [14], which can prevent NF-κB activation [15], treated simultaneously with prenatal LPS stimulation significantly attenuated prenatal LPS exposure-induced offspring’s renal damage [11]. These findings indicate that intra-renal NF-κB activation may have an important role in renal damage. However, PDTC simultaneously with LPS administration mainly blocks maternal NF-κB activation and inflammation response, which could only suggest that prenatal LPS exposure induced high levels of maternal inflammation may have a very important role in the development of renal damage in offspring. Thus, the physiological role of NF-κB activation in postnatal progressive renal damage in offspring of prenatal exposure to LPS is still uncertain.

Renal fibrosis is a common manifestation of various chronic kidney diseases, characterized by an excessive accumulation of extracellular matrix. The pathogenesis of renal fibrosis is a progressive process, which could ultimately lead to end-stage renal failure. In a simplistic view, renal fibrosis represents a failure of a wound-healing process in the kidney tissue responding to chronic, sustained injury [16]. We recently found that an early life and persistent NF-κB activation existed in the thoracic aorta [7] and increased renal collagen I expression [13] of prenatal LPS-induced offspring. Several researches showed that PDTC treatment could be a potential method to prevent renal damage in several animal models of chronic kidney diseases, such as doxorubicin hydrochloride induced nonimmune proteinuric tubulointerstitial inflammation [17], zymosan induced multiple organ failure [18], chronic tacrolimus (FK506) induced nephrotoxicity [19] and human renin and angiotensinogen genes double-transgenic rats [20]. As such, in the current study, we used PDTC to persistently inhibit the *in vivo* NF-κB activity of prenatal LPS-induced offspring postnatally in order to explore the role of NF-κB activation in the process of progressive renal fibrosis and its relationship with intra-renal RAS activation in offspring of prenatal LPS exposure.

## Material and Methods

### Animals

The study protocol was approved by the Ethical Committee for Animal Experimentation of Third Military Medical University. Nulliparous pregnant time-mated SD rats were obtained from the Experimental Animal Center of the Third Military Medical University (Chongqing, China). All animals had free access to standard laboratory rat chow and tap water. Until parturition, rats were housed individually in a room at constant temperature (24°C) and under a 12 hour light–dark cycle. The pups were raised with a lactating mother until 4 weeks of age, at which time they were removed to cages containing three or four pups. The present study conformed to the *Guide for the Care and Use of Laboratory Animals* published by the US National Institutes of Health (NIH Publication No. 85–23, revised 1996, revised 2011; www.nap.edu/catalog/5140.html).

The pregnant rats received an intraperitoneal (i.p) injection with saline (Control group) or 0.79 mg/kg LPS (Sigma Chemical, St. Louis, MO, USA) (LPS group), respectively, at gestational day 8, 10 and 12, as described previously (n = 8 for each group) [11, 21, 22]. The pregnant rats were used only once. After birth, the litter size was then reduced to eight pups to ensure equal nutrient access for all of the offspring. Neonatal rats were cared for by their mothers until they were weaned at the age of 4 weeks, after which they received standard rat chow. As both male and female offspring of prenatal exposure to LPS showed increased renal damage, equal size of male and female offspring rats were selected for each experiments in current study [11]. Offspring that received saline or LPS prenatally were treated with PDTC (120 mg/L, Sigma Chemical) [23, 24] through drinking water from postnatal 7 weeks to 20 or 68 weeks, defined as **Con+PDTC** or **LPS+PDTC** groups, respectively, by random selection. Offspring received saline or LPS prenatally without any additional post-natal treatment, identified as **Con+Ve** or **LPS+Ve** groups respectively. At the end of treatment, offspring were anesthetized using chloral hydrate (0.35 g/kg, 7% in saline) and sacrificed by decapitation. Kidneys were collected for future analysis.

During the period of treatment, the physical condition of all the animals was monitored every day. Some animals were dead prior to the experimental endpoint [S1 Fig], and some of which might be caused by long-term PDTC treatment. Once the animal showed reduced locomotive activity and any other weakness, such as arching, it will be supplied with mush food in the petri dish. Humane endpoints/early euthanasia with CO_2_ followed by cervical dislocation was used for animals who exhibited moribund states. The moribund states were identified as followings: weight loss (> 20%); inability to roll over from side to chest; dyspnea or labored breathing.

### Immunoblotting

Immunoblotting was performed as previously described [25, 26]. Briefly, kidney samples were lysed with T-PER^™^ tissue protein extraction reagent (Pierce, Rockford, IL) with protease inhibitor cocktail (Sigma-Aldrich, St. Loius, MO). The primary antibodies were as followings: NF-κB p65 (1:1000) and phospho-p65NF-κB (1:1000) (Cell signaling Technology, Beverly, MA, USA). GAPDH antibodies (1:5000, Cell Signaling Technology) was used as an internal control.

### Real-time RT-PCR

Total RNA was isolated by Trizol, and then was reverse transcribed using cDNA synthesis kit (DBI Bioscience, Ludwigshafen, Germany). All primers were designed by Premier 5.0 software or reported in previous literature [7], and were synthesized by Invitrogen. The primer sequences are as followings: rat *TNF-α* (sense: TGTTCATCCGTTCTCTACC; antisense: CCACTACTTCAGCGTCTC), rat *IL-6* (sense: CGGAGAGGAGACTTCACA; antisense: GCATCATCGCTGTTCATAC), rat *collagen type I* (*Col1a1*) (sense: ATCCTGCCGATGTCGCTAT; antisense: CCACAAGCGTGCTGTAGGT), rat *collagen type III* (*Col3a1*) (sense: CTGGTCCTGTTGGTCCATCT; antisense: ACCTTTGTCACCTCGTGGAC), rat angiotensin-converting enzyme (*ACE*) (sense: TTGGCTCTGTCTGTGTCT; antisense: CTCCTTGGTGATGCTTCC), rat *Ang* (sense: CTGGAGCTAAAGGACACACAGA; antisense: CAGGGTCTTCTCATCCACGG), rat *Renin* (sense: CACCTTCATCCGCAAGTT; antisense: GCAGAGCCAGACAGAATG), rat *AT1R* (sense: GGCAGGCACAGTTACATAT; antisense: CAAGGCGAGATTGAGAAGA). Each real-time PCR were carried out in a total volume of 20 μl with SYBR Green PCR Master Mix (DBI Bioscience) with the reaction condition: 95°C 2min, 40 cycles at 95°C 15S, 60°C 15S, 68°C 10S, 72°C 20S. Relative mRNA expression levels were firstly normalized to *β-actin* by using the ΔΔCt method and then normalized the value for each sample by divided it by the value of one sample from that of the Con+Ve group.

### Systolic blood pressure (SBP) measurement

SBP was assessed by the non-invasive tail-cuff method with computer-assisted BP-2010 Series tail measurement equipment (Softron Beijing Biotechnology Co., Ltd, Beijing, China), as described previously [6]. The investigators were blinded during the measuring of the blood pressure. SBP was calculated from three consecutive recordings.

### Ratio of kidney weight/body weight

Ratio of kidney weight/body weight was calculated by using the value of kidney weight divided by the value of whole body weight for each animal.

### Urine collection and analysis of 24 hour urine volume

Seven days ahead of sacrificing, each rat was housed in individual metabolic cage with free access to water and food for the collection of 24 hour urine. The mean value of 24 hour urine volume for each rat was calculated by using the 7 days consecutive data of urine volume.

### Histopathological evaluations

Renal tissue samples from offspring of 68 week-old were fixed with 4% para-formaldehyde, embedded in paraffin, and sectioned into 4-μm-thick sections. Hematoxylineosin (HE) staining was used to summarize the gross morphology of renal glomerulus, tubes and interstitium by using our standard in house protocol. Periodic acid-Schiff (PAS) stain was used for detecting saccharides, glomerulus basilar membrane and neutral mucus material by using PAS Kit, according to the manufacturer’s instructions (Sigma-Aldrich). Glomerular damage and tubulo-interstitial injury were semi-quantitatively scored, according to reported literature [27–29], by two independent examiners who were blinded as to animal groups. Briefly, randomly selected microscopic fields (6–8) at 100 × magnification per slide were used to count damaged glomerular or tubulo-interstitial injury.

Renal tissue samples from 68-week-old offspring were used for Sirius red staining. After de-waxing and re-hydration, slices were stained with Sirius red saturation picric acid for 30 minutes. After washed within water, nuclei were stained with hematoxylin. Slices were viewed with both light microscope (Olympus BH-2) and polarized microscope (Olympus BX-51). Collagen fibers appear as red under white microscope. Under polarized microscope, collagen I appears as yellow or red color with strong double refraction, while collagen III presents as green color with light double refraction.

The area of positive Sirius red staining was measured in 6–8 randomly chosen microscopic fields at 100 × magnification per offspring using NIH Image J software, according to the reported literature [30].

### Immunohistochemistry (IHC) staining of α-smooth muscle actin (α-SMA), Ang II and ACE

Renal α-SMA, AngII and ACE were identified by IHC as described previously [11, 31]. The antibodies were used as follows: α-SMA antibody (polyclone, Abcam, Cambridge, UK,1:2000 dilution), AngII antibody (polyclone, Abbiotec, CA, USA, 1:250 dilution) and ACE antibody (clone: 2E2, Abcam, 1:200 dilution).The positive area and density for α-SMA, Ang II and ACE in each group were semi-quantified using Image-pro plus software 5.1.

### Statistical analysis

For multiple comparisons, a two-way ANOVA model followed by LSD or Dunnett T3 test for inter-group comparison, when appropriated, was performed by PASW Statistic software 18.0. Data are shown as mean ± S.D. A P value less than 0.05 was considered statistically significant.

## Results

### Prenatal inflammatory exposure leads to NF-κB activation along with increased pro-inflammatory cytokines in renal tissue of offspring

**Fig 1:**
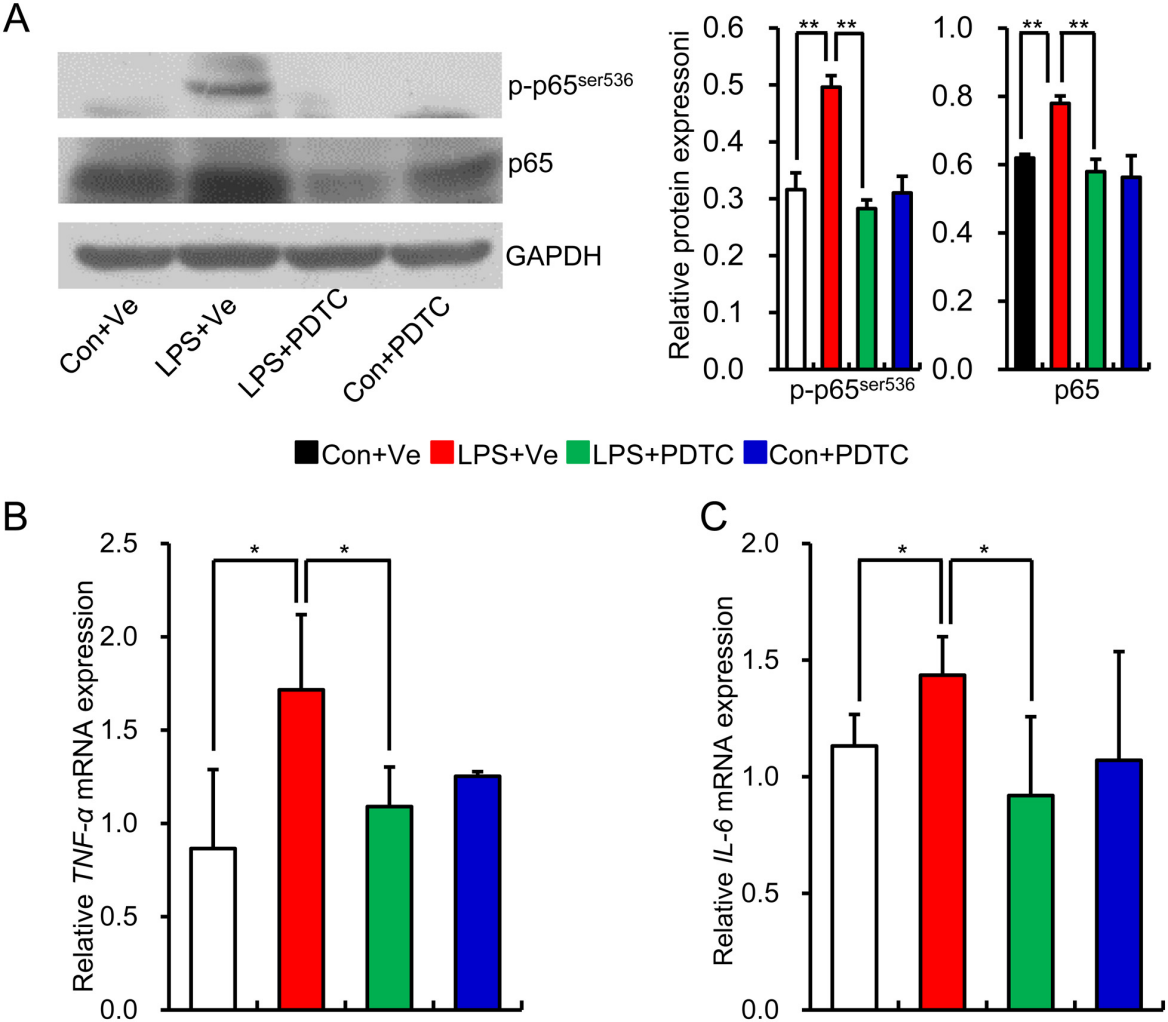
NF-κB activation and increased expression of pro-inflammatory cytokines existed in the renal tissue of offspring that received prenatal LPS exposure. Relative protein expression of phospho-p65^ser536^ and total p65 at the age of 20 weeks was determined by immunoblotting. Representative plots in each group and statistical data of relative densitometry, normalized by GAPDH, were shown (A). The mRNA expressions of *TNF-α* (B) and *IL-6* (C) in the kidney at the age of 20 weeks were determined by realtime RT-PCR. Data are presented as mean ± SD. n = 6 offspring per group for (A); n = 7 offspring in each group for (B) and (C). * and ** indicate P<0.05 and P<0.01, respectively, which denote statistical comparison between the two marked treatment groups (Two-way ANOVA for followed by Dunnett T3 test for inter-group comparison (A, B and C)). Con+Ve group, offspring rats from maternal saline treatment together with post-natal saline treatment; LPS+Ve group, offspring rats from maternal LPS exposure together with post-natal saline treatment; LPS+PDTC group, offspring rats from maternal LPS exposure together with post-natal PDTC treatment; Con+PDTC group, offspring rats from maternal saline treatment together post-natal PDTC treatment.

Our previously finding had shown that NF-κB activation existed in kidney of offspring that received prenatal LPS exposure at the age of 25 weeks [11]. We first assessed NF-κB activity by determining the protein level of p-p65 and p65 at the age of 20 weeks. The protein levels of p-p65 and total p65 were significantly increased in renal tissue of offspring that received prenatal exposure to LPS (Fig 1A), which indicates that intra-renal NF-κB was activated in adult offspring of prenatal exposure to LPS. Interestingly, post-natal administration of specific IκBα degradation inhibitor PDTC reversed the NF-κB activation, and was therefore consistent with our previous finding [11]. TNF*-α*, IL-1β and IL-6 are the main downstream pro-inflammatory cytokines of NF-κB activation [15, 32]. To find more evidence of NF-κB activation and *in vivo* effect of PDTC on NF-κB inhibition, we next determined the mRNA expression of *TNF-α*, and *IL-6* in renal tissue. We found that prenatal LPS exposure significantly increased the mRNA expression of *TNF-α* and *IL-6* in renal tissue of offspring at the age of 20 weeks, whereas post-natal PDTC administration significantly reduced the mRNA expression of *TNF-α* and *IL-6* (Fig 1B and 1C). This data demonstrates that NF-κB activation along with increased pro-inflammatory cytokines exists in the renal tissue of offspring that received prenatal exposure to LPS, whereas post-natal PDTC administration effectively blocks the NF-κB activation.

### Post-natal NF-κB inhibition represses offspring’s blood pressure elevation, increased kidney/body weight ratio and reduced urine volume (24 hour) induced by prenatal LPS exposure

**Fig 2:**
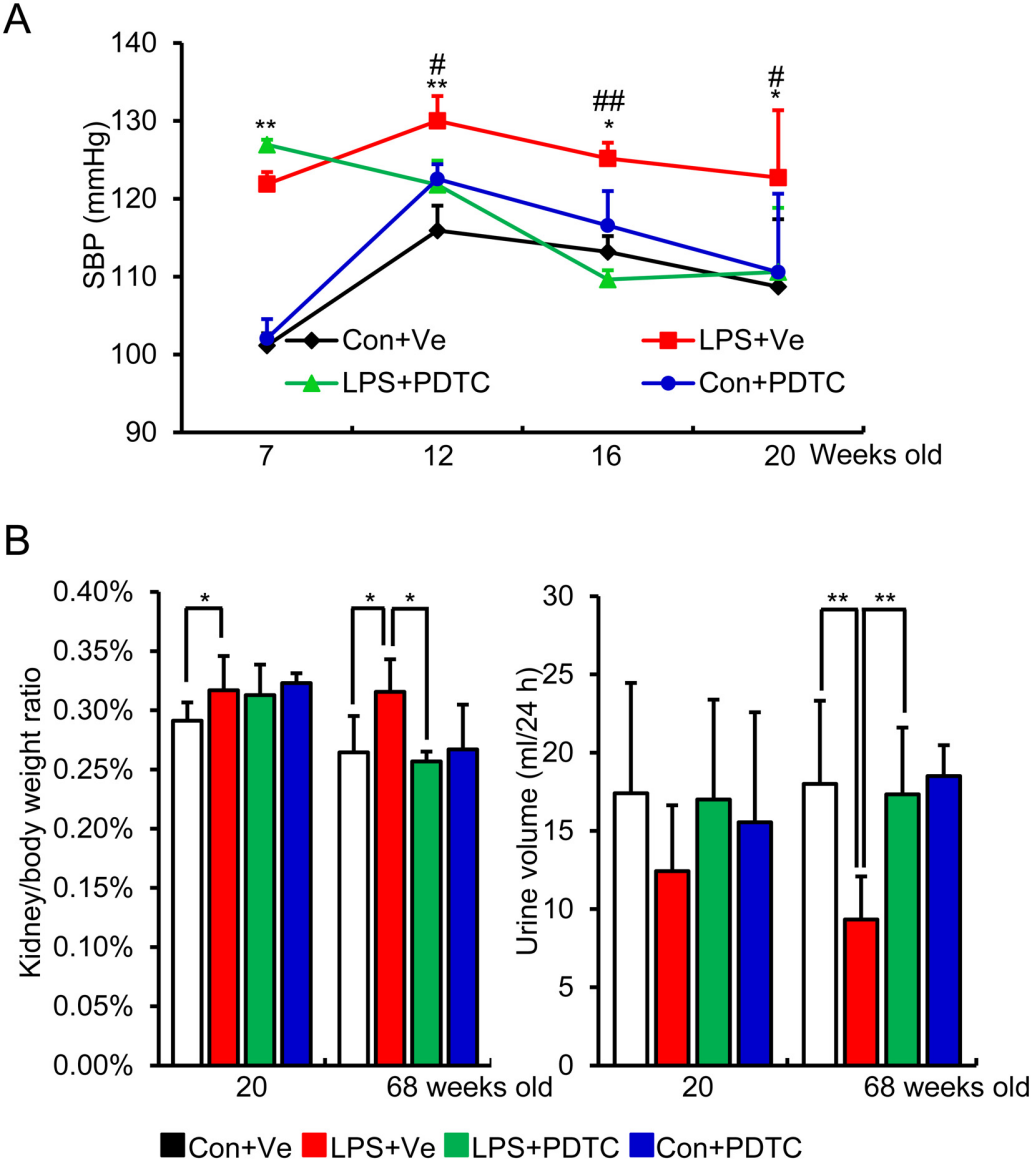
Post-natal inhibition of NF-κB activity by PDTC prevents blood pressure elevation (A), reverses the increased kidney/body weight ratio (B) and the reduced 24 hour urine volume (C). Systolic blood pressure (SBP) was measured by a noninvasive tail-cuff method at indicated time points after PDTC treatment (A). Relative kidney weight was calculated as a ratio of kidney wet weight to whole body weight after animals were sacrificed at indicated time-point (B). 24 hour urine volume was calculated by collecting urine for 24 hour by using metabolic cage at the indicated time-point (C). Data are presented as mean ± SD. n = 16 offspring for each group for (A, B, C).*P<0.05 or **P<0.01, Con+Ve vs LPS+Ve; #P<0.05 or ##P<0.01, LPS+PDTC vs LPS+Ve (A); * and ** indicate P<0.05 and P<0.01, respectively, which denote statistical comparison between the two marked treatment groups (B, C) (Two-way ANOVA followed by LSD test for inter-group comparison (A) or Dunnett T3 test (B and C) for inter-group comparison). Indications of Con+Ve, LPS+Ve, LPS+PDTC and Con+PDTC are as described in Fig 1.

Consistent with our previous findings, the current model also showed that offspring of prenatal exposure to LPS showed increased SBP, whereas post-natal PDTC treatment reversed this elevation (Fig 2A). Prenatal LPS exposure significantly increased offspring’s the ratio of kidney weight to whole body weight at the age of both 20 and 68 weeks, while post-natal PDTC treatment reversed this increment at the age of 68 weeks (Fig 2B, left panel). Since urine volume is one of the most direct indications of renal function, we also collected the urine by metabolic cage to assess the 24 hour urine volume. There were no significant changes in 24 hour urine volume among these groups at the age of 20 weeks. At the age of 68 weeks, the 24 hour urine volume was significantly reduced in offspring of prenatal exposure to LPS, whereas post-natal PDTC reversed it [Fig 2C]. Though this decreased 24 hour urine volume did not meet the diagnosing criterion of oliguria [33], it may be an indication of impaired kidney function in offspring of prenatal exposure to LPS, whereas post-natal NF-κB inhibition showed a protective effect.

### Prenatal LPS exposure induced offspring’s renal gross morphology damages could be reversed by post-natal NF-κB inhibition

**Fig 3:**
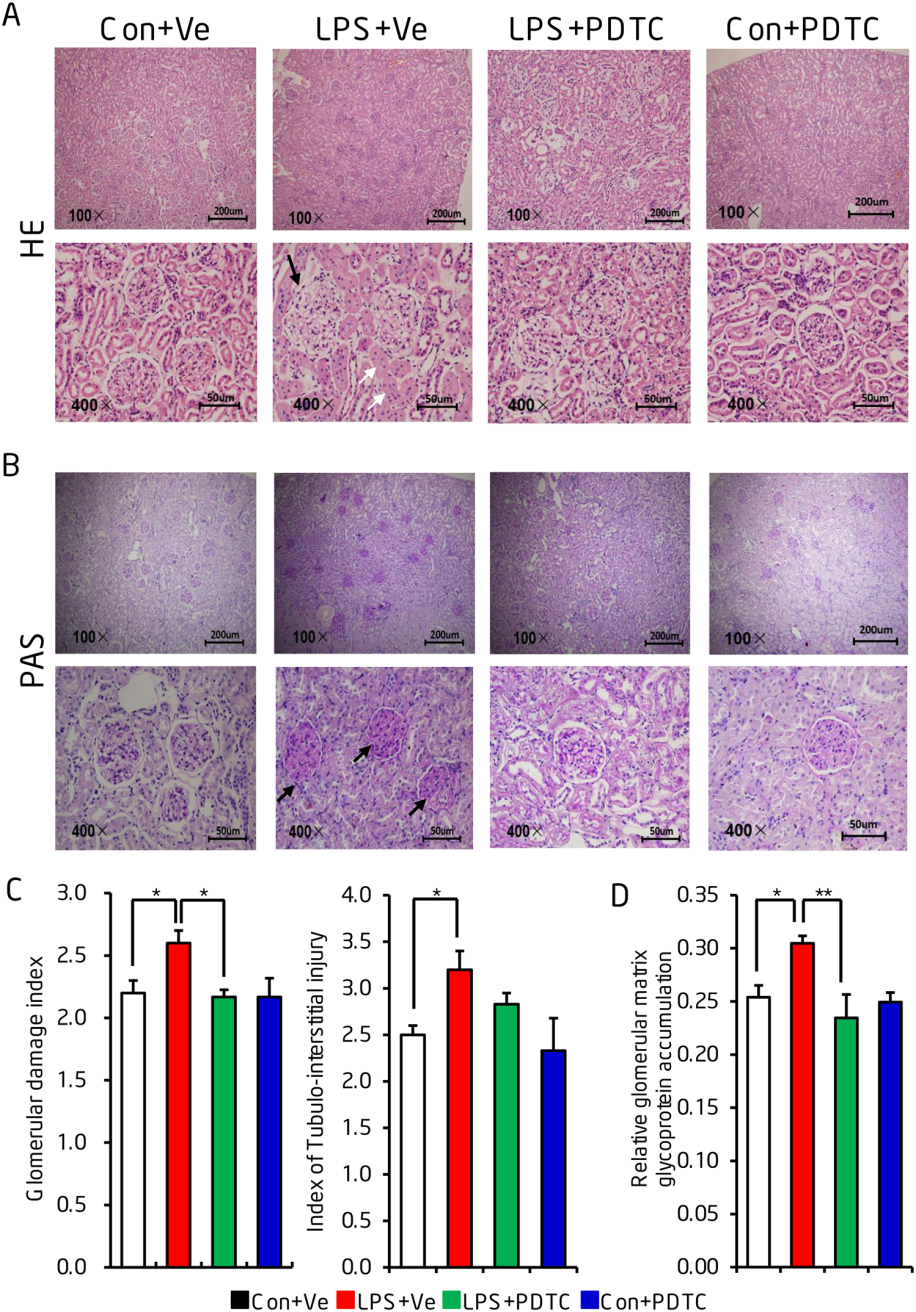
Post-natal NF-κB inhibition reverses the morphological changes in offspring of prenatal LPS exposure. Renal gross morphology was determined by Hematoxylin-eosin (HE) staining (A) and periodic acid–Schiff (PAS) staining (B) at the age of 68 weeks. Semi-quantitation of renal glomerular damage index and tubulo-interstitial injury index were semi-quantitatively from HE staining (C) and of relative glomerular matrix glycoprotein accumulation from PAS staining (D) were shown. The black arrows indicate obvious damaged glomerulus (A) or glomerulus with obvious mesangial matrix accumulation (B). The white arrows indicate the swelling of renal proximal tubules. Data are presented as mean ± SD. n = 8 offspring in each group for (A) and (B). n = 8 offspring and at least 100 glomeruli were counted from each offspring for (C) and (D). * and ** indicate P<0.05 and P<0.01, respectively, which denote statistical comparison between the two marked treatment groups (Two-way ANOVA followed by Dunnett T3 test (C) or LSD test (D) for inter-group comparison). Indications of Con+Ve, LPS+Ve, LPS+PDTC and Con+PDTC are as described in Fig 1.

HE staining of renal morphology at the age of 68 weeks showed an obvious increased infiltration of inflammatory cell, increased mesangial matrix fraction as well as the swelling of renal proximal tubules in the renal tissue of prenatal LPS-induced offspring when compared to that of control animals. This damage was prevented by post-natal PDTC administration (Fig 3A). PAS staining showed that offspring from prenatal LPS exposure presented with an increased mesangial matrix in glomeruli and also a few glomeruli undergoing sclerosis. This indicated increased glycoprotein accumulation in glomeruli. We also demonstrated that post-natal PDTC administration reversed this change [Fig 3B]. Semi-quantitation of glomerular damage and tubulo-interstitial injury further supported our idea that post-natal NF-κB activation plays an important role in progressive renal damage [Fig 3C and 3D].

### Inhibition of NF-κB activation protects offspring of prenatal LPS exposure from renal fibrosis

**Fig 4:**
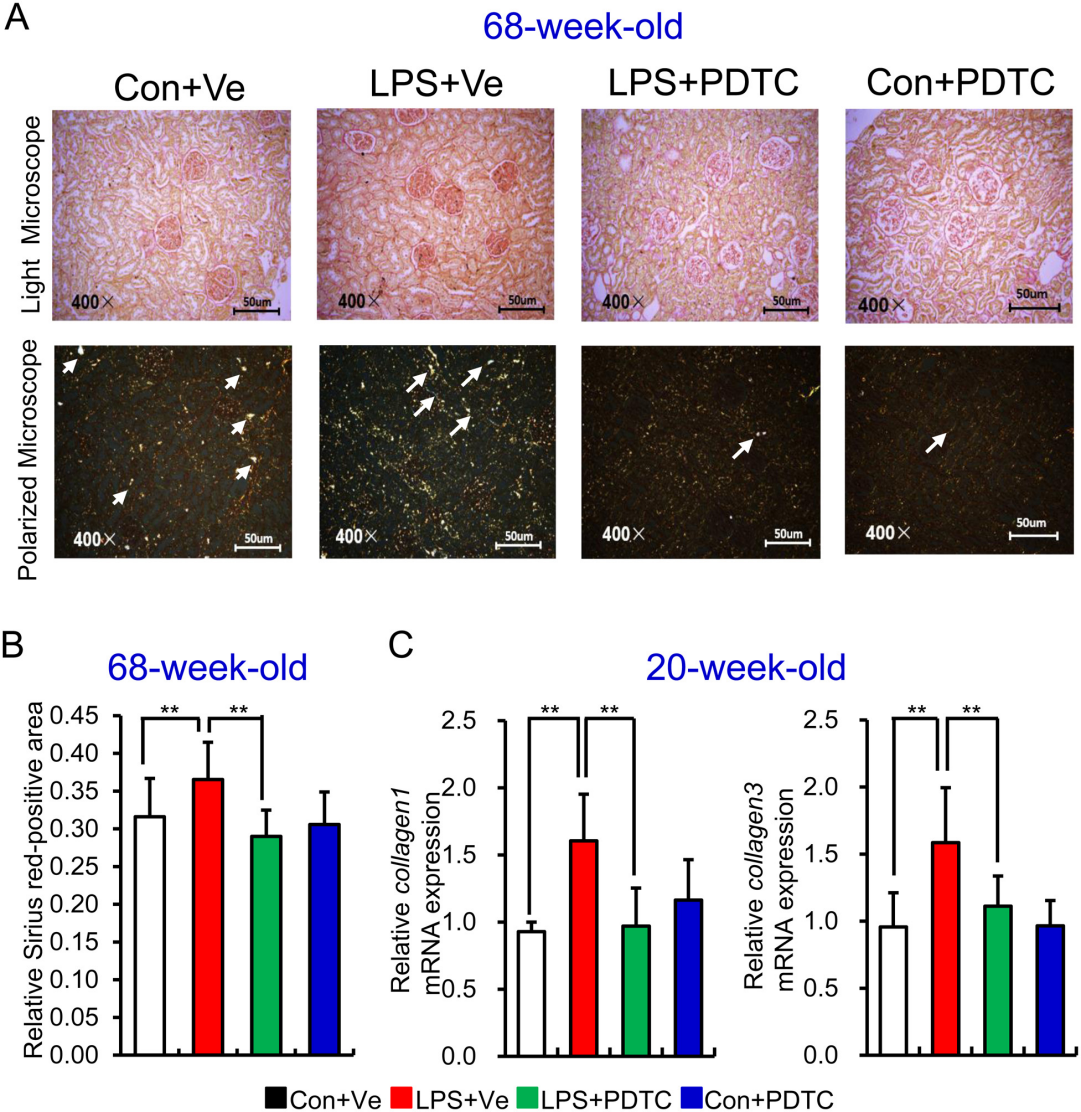
Renal collagen deposition in offspring of prenatal LPS exposure could be reversed by post-natal NF-κB inhibition. (A) Level of renal collagen deposition in 68-week-old offspring was assessed by Sirius red staining and observed under both white microscope (top panel) and polarized microscope (Arrow indicates collagen III accumulation, bottom panel). Semi-quantitation of relative Sirius red-positive area was shown (B). Relative mRNA expressions of *collagen type1* (*Col1a1*) and *collagen type 3* (*Col3a1*) (C) were determined by realtime RT-PCR in kidney at the age of 20 weeks. Data are presented as mean ± SD. n = 7 offspring and 4–5 pictures from each offspring were quantified for (B). n = 7 offspring in each group for (C). * and ** indicate P<0.05 and P<0.01, respectively, which denote statistical comparison between the two marked treatment groups (Two-way ANOVA followed by Dunnett T3 test (B) or LSD test (C) for inter-group comparison). Indications of Con+Ve, LPS+Ve, LPS+PDTC and Con+PDTC are as described in Fig 1.

Inflammation plays an important role in the progress of renal fibrosis [34]. As such we then explored the role of NF-κB activation in offspring’s renal fibrosis. We first determined renal fibrosis by Sirius red staining and viewed the slices under both light microscope (Fig 4A, top panel) and polarized microscope (Fig 4A, bottom panel). Our results showed that prenatal LPS exposure led to increased Sirius red positive area, which was mainly type III collagen fibers in renal tissue of offspring at the age of 68 weeks, whereas post-natal NF-κB inhibition by PDTC reversed this damage **[**Fig 4A and 4B**]**. Analysis of the mRNA expression of *collagen 1* and *collagen 3* at an earlier age of 20 weeks showed that renal *collagen 1* and *collagen 3* transcripts were significantly increased in offspring of prenatal exposure to LPS, whereas post-natal PDTC treatment repressed these increments [Fig 4C].

**Fig 5:**
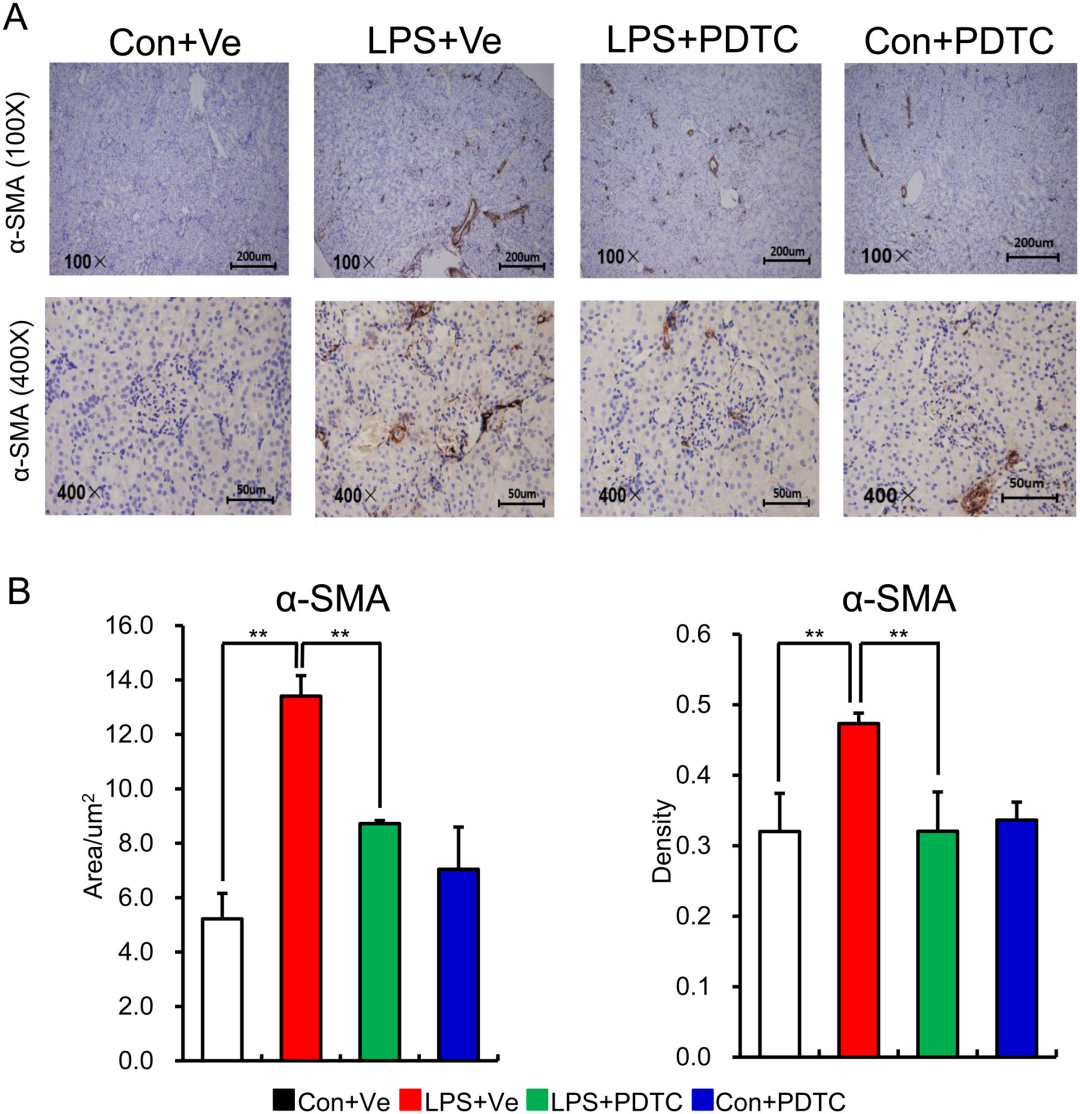
Increased protein level of intrarenal α-SMA expression in offspring of prenatal LPS exposure could be reversed by post-natal NF-κB inhibition. Renal α-SMA expression in offspring at the age of 68 weeks was determined by immunohistochemistry (A) and semi-quantitation of its positive area and density was show in (B). Data are presented as mean ± SD. n = 7 offspring and 4–5 pictures from each offspring were quantified for (B). ** indicates P<0.01, which denotes statistical comparison between the two marked treatment groups (Two-way ANOVA followed by LSD test (B) for inter-group comparison). Indications of Con+Ve, LPS+Ve, LPS+PDTC and Con+PDTC are as described in Fig 1.

α-SMA, predominantly existing in vasculature [35], is the actin isoform that plays an important role in fibrogenesis [36]. In health kidney, α-SMA is only expressed in the middle or adventitia layer of vasculature, while it occurs at the renal interstitial during renal fibrosis [37, 38]. During the medical condition of glomerular nephritis, inflammatory cells in glomerular and renal interstitial can be activated, leading to an increase in actin synthesis (indicated by the expression of α-SMA). As such, inflammatory injuries can be aggravated leading to further fibrogenesis of the kidney [39, 40]. To find more evidence of renal fibrosis in offspring of prenatal LPS exposure, we next determined the α-SMA expression in renal tissue by IHC. Our data showed that little cells in kidney expressed α-SMA in control offspring at the age of 68 weeks, whereas much higher level of α-SMA appeared at the interstitial in offspring of prenatal LPS exposure. As expected, post-natal PDTC administration obviously prevented the increased expression of α-SMA in the interstitial of renal tissue (Fig 5). All this data above suggests that post-natal NF-κB activation along with pro-inflammatory status has a critical role in the process of gradual interstitial renal fibrosis and post-natal NF-κB inhibition could be a potential therapeutic target for renal damage of prenatal inflammation stimulated offspring.

### RAS over-activity could be reversed by post-natal NF-κB inhibition in offspring of prenatal LPS exposure

**Fig 6:**
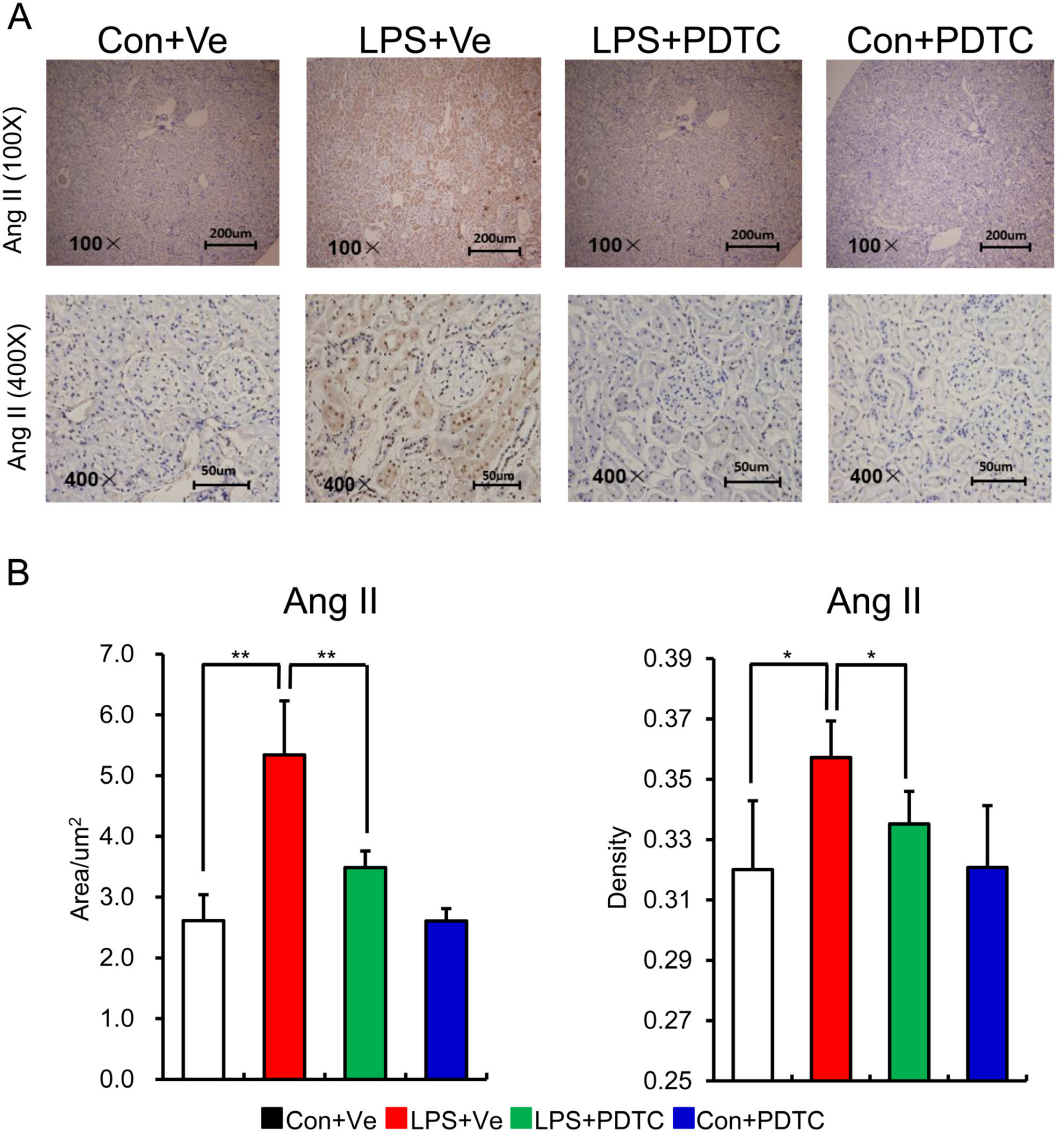
Higher level of Ang II positive cell in kidney in offspring of prenatal LPS exposure is reversed by post-natal NF-κB inhibition. The protein expression of Ang II in renal tissue of offspring at the age of 20 weeks was determined by immunohistochemistry staining. Representative picture from each group (A) and semi-quantitation of its positive area and density (B) are shown. Data are shown as mean ± SD. n = 7 offspring and 4–5 pictures from each offspring were quantified for (B). * and ** indicate P<0.05 and P<0.01, respectively, which denote statistical comparison between the two marked treatment groups (Two-way ANOVA followed by LSD test (B) for inter-group comparison). Indications of Con+Ve, LPS+Ve, LPS+PDTC and Con+PDTC are as described in Fig 1.

**Fig 7:**
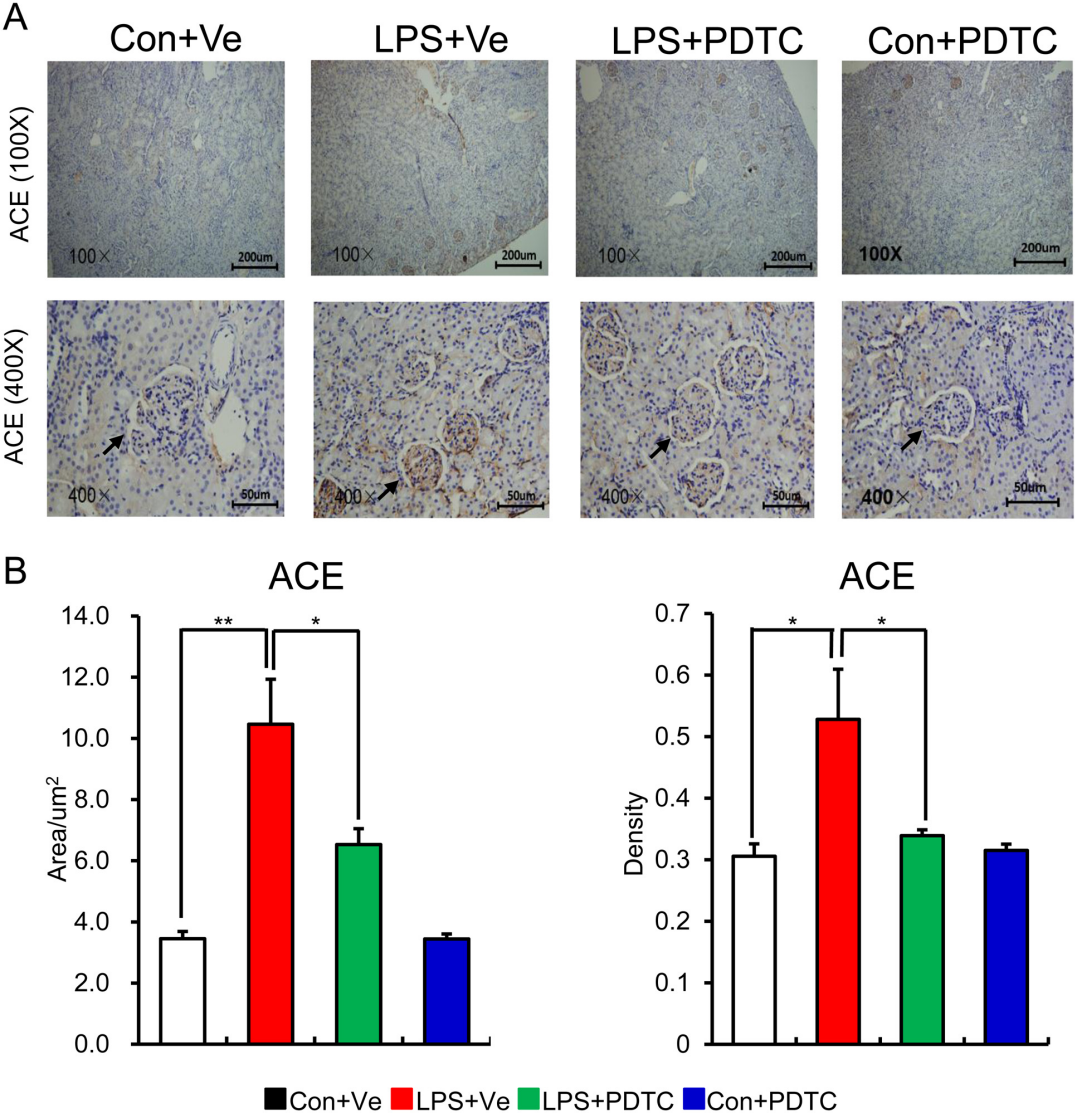
Post-natal NF-κB inhibition protects offspring of prenatal LPS exposure from ACE overexpression in kidney. The protein level of ACE in kidney of offspring at the age of 20 weeks was assessed by immunohistochemistry staining. Representative picture from each group (A) and semi-quantitation of its positive area and density (B) are shown. Data are presented as mean ± SD. n = 7 offspring and 4–5 pictures from each offspring were quantified for (B). * and ** indicate P<0.05 and P<0.01, respectively, which denote statistical comparison between the two marked treatment groups (Two-way ANOVA followed by LSD test (B) for inter-group comparison). Indications of Con+Ve, LPS+Ve, LPS+PDTC and Con+PDTC are as described in Fig 1.

**Fig 8:**
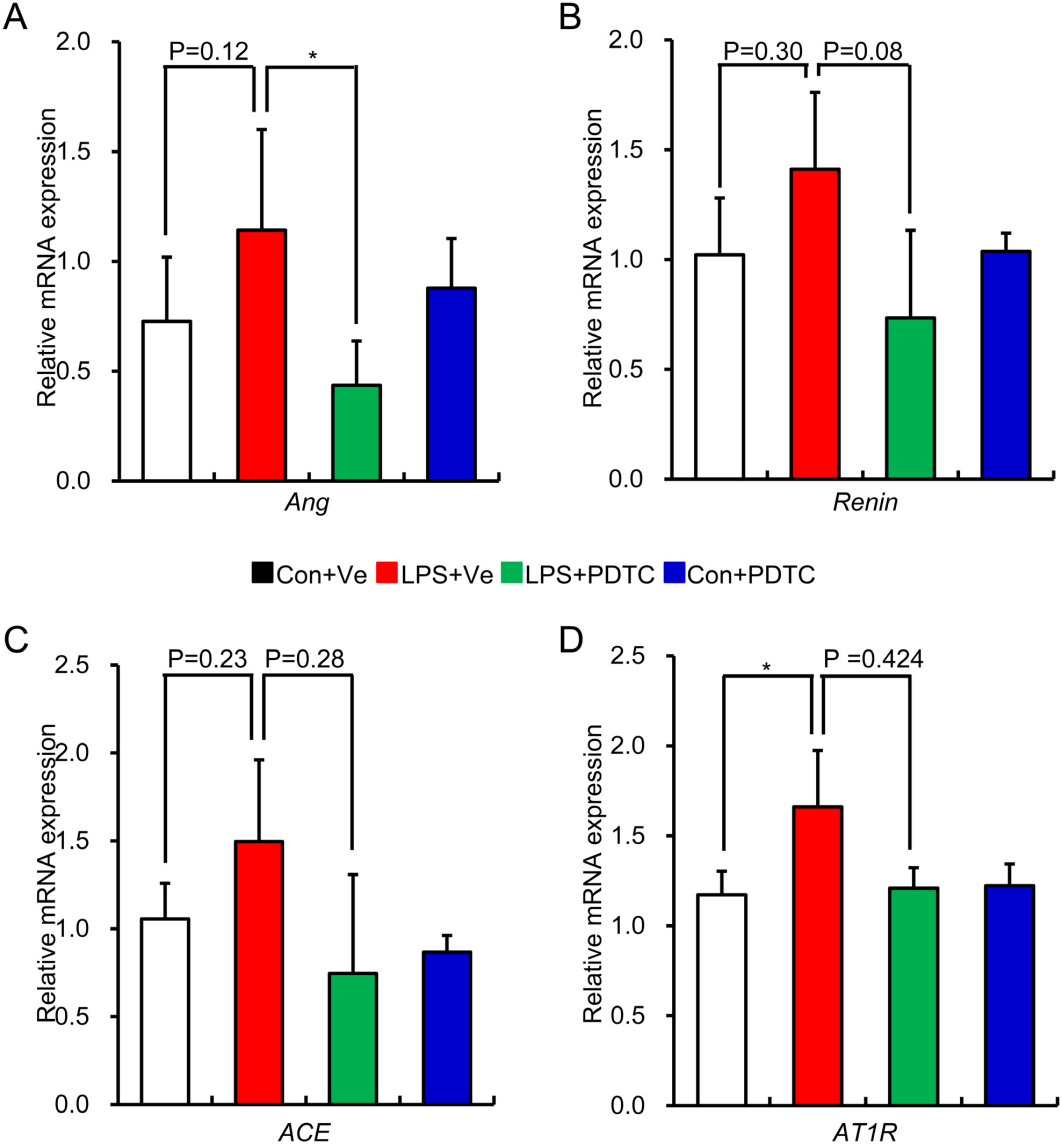
The effect of Post-natal NF-κB inhibition on aberrant renal RAS gene expression in offspring of prenatal LPS exposure. Relative mRNA expression of renal *Angiotensinogen* (A), *Renin* (B), *ACE* (C) and *AT1R* (D) in offspring at the age of 20 weeks was determined by real time RT-PCR. Data are shown as mean ± SD. n = 7 in each group. * indicates P<0.05, which denotes statistical comparison between the two marked treatment groups (Two-way ANOVA followed by Dunnett T3 test (A,B,C, D) for inter-group comparison). Indications of Con+Ve, LPS+Ve, LPS+PDTC and Con+PDTC are as described in Fig 1.

Inflammation leads to over activation of RAS, while enhanced RAS activity positively aggravates inflammation response in local tissue through NF-κB signal pathway. This positive feedback cycle is critical in causing tissue damage [41]. In addition, the cross-talk among RAS, NF-κB and pro-inflammatory cytokines obviously exacerbates the progress of renal fibrosis [42]. So we next detected the role of post-natal NF-κB inhibition by PDTC on renal RAS activity of prenatal LPS exposed offspring. As expected, post-natal PDTC treatment restored the increased protein expression of renal Ang II and ACE in offspring of prenatal LPS exposure [Figs 6 and 7]. Unexpectedly, the mRNA expression of RAS family components showed the similar trend as the protein expressions without statistical significance [Fig 8]. This might due to the large variations of the samples or the limited sample size for each group.

## Discussion

Inflammation plays a critical role in the process of progressive renal malfunction, fibrosis and chronic renal failure [43]. However, the exact mechanisms for initiation and development of persistent inflammation status are largely unknown. Our research laboratory previously found that prenatal inflammatory exposure led to higher level of pro-inflammatory cytokines in fetus, renal damage in newborns [44] and NF-κB activation in adult [11]. Our current study showed that intra-renal elevated levels of pro-inflammatory cytokines, such as TNF-α and IL-6, were found in offspring that were exposed to an inflammatory stimuli during the prenatal period. All these findings suggest that prenatal inflammatory exposure should be a momentous but also easily ignored etiology of chronic renal inflammation. As such, prevention of inflammation during earlier life may have a great significance on reducing the incidence of several kidney diseases related to chronic renal inflammation, such as renal malfunction and renal failure.

Previous studies have demonstrated that higher levels of inflammatory activity are positively correlated with renal fibrosis and renal hypo-function [45]. In this study, we show that prenatal inflammatory exposure led to obvious offspring’s intra-renal damage, such as increased infiltration of inflammatory cell and mesangial matrix fraction, as well as accumulation of glycogen in both glomeruli and interstitial area. Post-natal NF-κB inhibition, through daily drinking water with PDTC, strikingly repressed the expression level of pro-inflammatory cytokines together with obvious protection of progressive kidney damage. As such, our results suggest that NF-κB-dependent persistent inflammatory activation might drive the progressive pathology of renal damage in offspring of prenatal inflammatory exposure. Previous findings showed that prenatal inflammatory exposure led to increased collagen I expression [13], which was significantly increased when renal fibrosis occurred. Our current study provides additional supportive in regard to our finding of intra-renal fibrosis as evident by increased expression of collagen III, α-SMA and collagen hyperplasia in kidney of prenatal LPS-induced offspring. All these changes related to renal fibrosis were reversed by post-natal NF-κB inhibition. Together with our previously finding that prenatal inflammatory exposure directly led to reduced total number of glomeruli during the developmental stage [11, 13], our results suggest that prenatal inflammatory stimulus cause both direct micro-structural damage and proneness to activation of inflammatory response. In the adult, these two factors cross-talk with each other, which in turn aggravate and worsen the resultant renal damage.

Mechanistically, we found that the protective effects of NF-κB inhibition on renal fibrosis in offspring of prenatal LPS exposure might be attributed to repressed RAS over-activity. The RAS is described as a cascade of biochemical reactions, whose activity is essential for cardiovascular homeostasis [46]. Cross-talk of pro-inflammatory status and RAS over-activation plays a critical role in progressive renal fibrosis [47, 48]. We previously found that prenatal LPS exposure led to decreased renal cortex renin and Ang II expression in offspring at 1 day of age, but significantly increased at 7, 16 and 25 weeks [11]. Our current finding showed that post-natal PDTC treatment significantly reduced the intra-renal expression level of ACE and Ang II in offspring exposed to prenatal inflammatory stimulus. All these findings suggests that the cross-talk between RAS abnormality and NF-κB activation interact with each other thereby accelerating the progressive renal damage in offspring of prenatal exposure to LPS.

In summary, the activation of the offspring’s intra-renal NF-κB activation caused by prenatal inflammatory exposure cross-talks with excessive RAS activation, exaggerates offspring’s renal inflammation status, renal fibrosis and further damage of renal function. Thus, early life prevention of excessive NF-κB activation may be a potential preventive strategy for chronic renal inflammation and progressive renal injury.

## Supporting Information

S1 Fig Kaplan–Meier survival curve of offspring that received saline or prenatal LPS exposure together with post-natal PDTC treatment. Con+Ve group, offspring rats from maternal saline treatment together with post-natal saline treatment; LPS+Ve group, offspring rats from maternal LPS exposure together with post-natal saline treatment; LPS+PDTC group, offspring rats from maternal LPS exposure together with post-natal PDTC treatment; Con+PDTC group, offspring rats from maternal saline treatment together post-natal PDTC treatment.
(TIF)

## References

[1] Chow CK, Teo KK, Rangarajan S, Islam S, Gupta R, Avezum A, et al Prevalence, awareness, treatment, and control of hypertension in rural and urban communities in high-, middle-, and low-income countries. JAMA. 2013;310(9):959–68. 10.1001/jama.2013.184182 24002282

[2] Palinski W. Effect of maternal cardiovascular conditions and risk factors on offspring cardiovascular disease. Circulation. 2014;129(20):2066–77. 10.1161/CIRCULATIONAHA.113.001805 24842934 PMC4053195

[3] Hay PE, Lamont RF, Taylor-Robinson D, Morgan DJ, Ison C, Pearson J. Abnormal bacterial colonisation of the genital tract and subsequent preterm delivery and late miscarriage. BMJ. 1994;308(6924):295–8. 8124116 10.1136/bmj.308.6924.295 PMC2539287

[4] Arends NJ, Boonstra VH, Duivenvoorden HJ, Hofman PL, Cutfield WS, Hokken-Koelega AC. Reduced insulin sensitivity and the presence of cardiovascular risk factors in short prepubertal children born small for gestational age (SGA). Clin Endocrinol (Oxf). 2005;62(1):44–50. 10.1111/j.1365-2265.2004.02171.x 15638869

[5] Lien E, Means TK, Heine H, Yoshimura A, Kusumoto S, Fukase K, et al Toll-like receptor 4 imparts ligand-specific recognition of bacterial lipopolysaccharide. J Clin Invest. 2000;105(4):497–504. 10.1172/JCI8541 10683379 PMC289161

[6] Wei YL, Li XH, Zhou JZ. Prenatal exposure to lipopolysaccharide results in increases in blood pressure and body weight in rats. Acta Pharmacol Sin. 2007;28(5):651–6. 10.1111/j.1745-7254.2007.00593.x 17439721

[7] Deng Y, Deng Y, He X, Chu J, Zhou J, Zhang Q, et al Prenatal inflammation-induced NF-kappaB dyshomeostasis contributes to renin-angiotensin system over-activity resulting in prenatally programmed hypertension in offspring. Sci Rep. 2016;6:21692 10.1038/srep21692 26877256 PMC4753429

[8] Gorgas DL. Infections related to pregnancy. Emergency medicine clinics of North America. 2008;26(2):345–66, viii 10.1016/j.emc.2008.01.007 18406978

[9] Safir A, Levy A, Sikuler E, Sheiner E. Maternal hepatitis B virus or hepatitis C virus carrier status as an independent risk factor for adverse perinatal outcome. Liver Int. 2010;30(5):765–70. 10.1111/j.1478-3231.2010.02218.x 20214739

[10] de Steenwinkel FD, Hokken-Koelega AC, de Man YA, de Rijke YB, de Ridder MA, Hazes JM, et al Circulating maternal cytokines influence fetal growth in pregnant women with rheumatoid arthritis. Annals of the rheumatic diseases. 2013;72(12):1995–2001. 10.1136/annrheumdis-2012-202539 23264340

[11] Hao XQ, Zhang HG, Yuan ZB, Yang DL, Hao LY, Li XH. Prenatal exposure to lipopolysaccharide alters the intrarenal renin-angiotensin system and renal damage in offspring rats. Hypertens Res. 2010;33(1):76–82. 10.1038/hr.2009.185 19911002

[12] Wang X, Luo H, Chen C, Chen K, Wang J, Cai Y, et al Prenatal lipopolysaccharide exposure results in dysfunction of the renal dopamine D1 receptor in offspring. Free Radic Biol Med. 2014;76:242–50. 10.1016/j.freeradbiomed.2014.08.010 25236748 PMC6873924

[13] Hao XQ, Kong T, Zhang SY, Zhao ZS. Alteration of embryonic AT(2)-R and inflammatory cytokines gene expression induced by prenatal exposure to lipopolysaccharide affects renal development. Experimental and toxicologic pathology: official journal of the Gesellschaft fur Toxikologische Pathologie. 2013;65(4):433–9. 10.1016/j.etp.2012.01.001 22342485

[14] Schreck R, Meier B, Mannel DN, Droge W, Baeuerle PA. Dithiocarbamates as potent inhibitors of nuclear factor kappa B activation in intact cells. The Journal of experimental medicine. 1992;175(5):1181–94. 1314883 10.1084/jem.175.5.1181 PMC2119220

[15] Liu SF, Ye X, Malik AB. Inhibition of NF-kappaB activation by pyrrolidine dithiocarbamate prevents In vivo expression of proinflammatory genes. Circulation. 1999;100(12):1330–7. 10491379 10.1161/01.cir.100.12.1330

[16] Liu Y. Renal fibrosis: new insights into the pathogenesis and therapeutics. Kidney Int. 2006;69(2):213–7. 10.1038/sj.ki.5000054 16408108

[17] Rangan GK, Wang Y, Tay YC, Harris DC. Inhibition of nuclear factor-kappaB activation reduces cortical tubulointerstitial injury in proteinuric rats. Kidney Int. 1999;56(1):118–34. 10.1046/j.1523-1755.1999.00529.x 10411685

[18] Cuzzocrea S, Rossi A, Pisano B, Di Paola R, Genovese T, Patel NS, et al Pyrrolidine dithiocarbamate attenuates the development of organ failure induced by zymosan in mice. Intensive care medicine. 2003;29(11):2016–25. 10.1007/s00134-003-1887-8 12879239

[19] Tamada S, Nakatani T, Asai T, Tashiro K, Komiya T, Sumi T, et al Inhibition of nuclear factor-kappaB activation by pyrrolidine dithiocarbamate prevents chronic FK506 nephropathy. Kidney Int. 2003;63(1):306–14. 10.1046/j.1523-1755.2003.00714.x 12472797

[20] Muller DN, Dechend R, Mervaala EM, Park JK, Schmidt F, Fiebeler A, et al NF-kappaB inhibition ameliorates angiotensin II-induced inflammatory damage in rats. Hypertension. 2000;35(1 Pt 2):193–201. 10.1161/01.HYP.35.1.193 10642297

[21] Nilsson C, Larsson BM, Jennische E, Eriksson E, Bjorntorp P, York DA, et al Maternal endotoxemia results in obesity and insulin resistance in adult male offspring. Endocrinology. 2001;142(6):2622–30. 10.1210/endo.142.6.8191 11356713

[22] Ornoy A, Altshuler G. Maternal endotoxemia, fetal anomalies, and central nervous system damage: a rat model of a human problem. Am J Obstet Gynecol. 1976;124(2):196–204. 1108657 10.1016/s0002-9378(16)33298-7

[23] Elks CM, Mariappan N, Haque M, Guggilam A, Majid DS, Francis J. Chronic NF-{kappa}B blockade reduces cytosolic and mitochondrial oxidative stress and attenuates renal injury and hypertension in SHR. Am J Physiol Renal Physiol. 2009;296(2):F298–305. 10.1152/ajprenal.90628.2008 19073636 PMC2643866

[24] Koeners MP, Braam B, Joles JA. Perinatal inhibition of NF-kappaB has long-term antihypertensive effects in spontaneously hypertensive rats. J Hypertens. 2011;29(6):1160–6. 10.1097/HJH.0b013e3283468344 21505356

[25] Deng Y, Kerdiles Y, Chu J, Yuan S, Wang Y, Chen X, et al Transcription factor foxo1 is a negative regulator of natural killer cell maturation and function. Immunity. 2015;42(3):457–70. 10.1016/j.immuni.2015.02.006 25769609 PMC4400836

[26] Deng Y, Chu J, Ren Y, Fan Z, Ji X, Mundy-Bosse B, et al The Natural Product Phyllanthusmin C Enhances IFN-gamma Production by Human NK Cells through Upregulation of TLR-Mediated NF-kappaB Signaling. J Immunol. 2014;193(6):2994–3002. 10.4049/jimmunol.1302600 25122922 PMC4162489

[27] Elbe H, Vardi N, Esrefoglu M, Ates B, Yologlu S, Taskapan C. Amelioration of streptozotocin-induced diabetic nephropathy by melatonin, quercetin, and resveratrol in rats. Hum Exp Toxicol. 2015;34(1):100–13. 10.1177/0960327114531995 24812155

[28] Wang Z, Zhu Q, Li PL, Dhaduk R, Zhang F, Gehr TW, et al Silencing of hypoxia-inducible factor-1alpha gene attenuates chronic ischemic renal injury in two-kidney, one-clip rats. Am J Physiol Renal Physiol. 2014;306(10):F1236–42. 10.1152/ajprenal.00673.2013 24623146 PMC4024731

[29] Raij L, Azar S, Keane W. Mesangial immune injury, hypertension, and progressive glomerular damage in Dahl rats. Kidney Int. 1984;26(2):137–43. 10.1038/ki.1984.147 6239058

[30] Kim J, Imig JD, Yang J, Hammock BD, Padanilam BJ. Inhibition of soluble epoxide hydrolase prevents renal interstitial fibrosis and inflammation. Am J Physiol Renal Physiol. 2014;307(8):F971–80. 10.1152/ajprenal.00256.2014 25164080 PMC4200297

[31] Zhao S, Zhang H, Cao D, Liu Y, Li X. Lipopolysaccharide exposure during pregnancy leads to aortic dysfunction in offspring rats. PLoS One. 2014;9(7):e102273 10.1371/journal.pone.0102273 25025169 PMC4099131

[32] Lin TH, Tamaki Y, Pajarinen J, Waters HA, Woo DK, Yao Z, et al Chronic inflammation in biomaterial-induced periprosthetic osteolysis: NF-kappaB as a therapeutic target. Acta Biomater. 2014;10(1):1–10. 10.1016/j.actbio.2013.09.034 24090989 PMC3849197

[33] Bagshaw SM, Bellomo R, Kellum JA. Oliguria, volume overload, and loop diuretics. Critical care medicine. 2008;36(4 Suppl):S172–8. 10.1097/CCM.0b013e318168c92f 18382190

[34] Blank U, Essig M, Scandiuzzi L, Benhamou M, Kanamaru Y. Mast cells and inflammatory kidney disease. Immunological reviews. 2007;217:79–95. 10.1111/j.1600-065X.2007.00503.x 17498053

[35] Lazarides E, Burridge K. Alpha-actinin: immunofluorescent localization of a muscle structural protein in nonmuscle cells. Cell. 1975;6(3):289–98. 10.1016/0092-8674(75)90180-4 802682

[36] Kawasaki Y, Imaizumi T, Matsuura H, Ohara S, Takano K, Suyama K, et al Renal expression of alpha-smooth muscle actin and c-Met in children with Henoch-Schonlein purpura nephritis. Pediatr Nephrol. 2008;23(6):913–9. 10.1007/s00467-008-0749-6 18273647

[37] Boukhalfa G, Desmouliere A, Rondeau E, Gabbiani G, Sraer JD. Relationship between alpha-smooth muscle actin expression and fibrotic changes in human kidney. Experimental nephrology. 1996;4(4):241–7. 8864727

[38] Roson MI, Cavallero S, Della Penna S, Cao G, Gorzalczany S, Pandolfo M, et al Acute sodium overload produces renal tubulointerstitial inflammation in normal rats. Kidney Int. 2006;70(8):1439–46. 10.1038/sj.ki.5001831 16955102

[39] Noronha IL, Fujihara CK, Zatz R. The inflammatory component in progressive renal disease—are interventions possible? Nephrol Dial Transplant. 2002;17(3):363–8. 10.1093/ndt/17.3.363 11865077

[40] Boor P, Ostendorf T, Floege J. Renal fibrosis: novel insights into mechanisms and therapeutic targets. Nat Rev Nephrol. 2010;6(11):643–56. 10.1038/nrneph.2010.120 20838416

[41] Benigni A, Cassis P, Remuzzi G. Angiotensin II revisited: new roles in inflammation, immunology and aging. EMBO molecular medicine. 2010;2(7):247–57. 10.1002/emmm.201000080 20597104 PMC3377325

[42] Klahr S, Morrissey JJ. The role of vasoactive compounds, growth factors and cytokines in the progression of renal disease. Kidney international Supplement. 2000;75(S75):S7–14. 10.1046/j.1523-1755.2000.07509.x 10828755

[43] Kurts C, Panzer U, Anders HJ, Rees AJ. The immune system and kidney disease: basic concepts and clinical implications. Nature reviews Immunology. 2013;13(10):738–53. 10.1038/nri3523 24037418

[44] Zhou J, Zhang X, Zhang H, Jia Y, Liu Y, Tang Y, et al Use of data mining to determine changes in the gene expression profiles of rat embryos following prenatal exposure to inflammatory stimulants. Mol Med Rep. 2013;8(1):95–102. 10.3892/mmr.2013.1498 23722200

[45] Liu Y. Cellular and molecular mechanisms of renal fibrosis. Nat Rev Nephrol. 2011;7(12):684–96. 10.1038/nrneph.2011.149 22009250 PMC4520424

[46] Rosenbaugh EG, Savalia KK, Manickam DS, Zimmerman MC. Antioxidant-based therapies for angiotensin II-associated cardiovascular diseases. Am J Physiol Regul Integr Comp Physiol. 2013;304(11):R917–28. 10.1152/ajpregu.00395.2012 23552499 PMC3680755

[47] Mezzano SA, Ruiz-Ortega M, Egido J. Angiotensin II and renal fibrosis. Hypertension. 2001;38(3 Pt 2):635–8. 10.1161/hy09t1.094234 11566946

[48] Ruiz-Ortega M, Ruperez M, Esteban V, Rodriguez-Vita J, Sanchez-Lopez E, Carvajal G, et al Angiotensin II: a key factor in the inflammatory and fibrotic response in kidney diseases. Nephrol Dial Transplant. 2006;21(1):16–20. 10.1093/ndt/gfi265 16280370

