# Supplementary figures and images for: Post-Natal Inhibition of NF-κB Activation Prevents Renal Damage Caused by Prenatal LPS Exposure
Source: PLoS One. 2016 Apr 13;11(4):e0153434. doi: 10.1371/journal.pone.0153434 (PMC4830567; doi:10.1371/journal.pone.0153434)

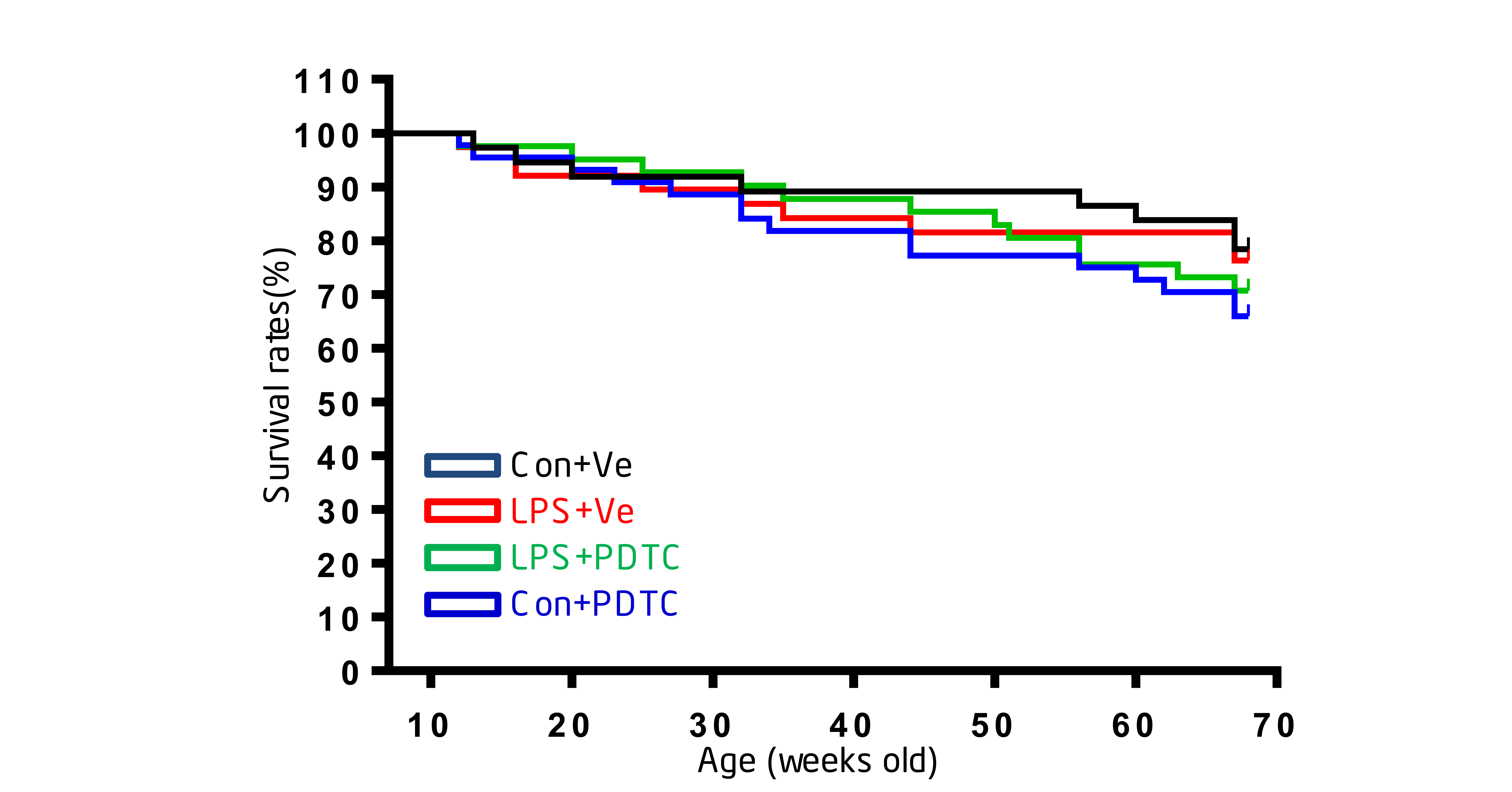

Supplement: S1 Fig — Con+Ve group, offspring rats from maternal saline treatment together with post-natal saline treatment; LPS+Ve group, offspring rats from maternal LPS exposure together with post-natal saline treatment; LPS+PDTC group, offspring rats from maternal LPS exposure together with post-natal PDTC treatment; Con+PDTC group, offspring rats from maternal saline treatment together post-natal PDTC treatment. (TIF) [file pone.0153434.s001.tif]
